# A Fiber Bragg Grating Sensing-Based Micro-Vibration Sensor and Its Application

**DOI:** 10.3390/s16040547

**Published:** 2016-04-15

**Authors:** Tianliang Li, Yuegang Tan, Zude Zhou

**Affiliations:** School of Mechanical and Electronic Engineering, Wuhan University of Technology, Wuhan 430000, Hubei, China; ygtan@whut.edu.cn (Y.T.); zudezhou@whut.edu.cn (Z.Z.)

**Keywords:** micro-vibration, fiber Bragg grating, vibration sensor

## Abstract

This paper proposes a fiber Bragg grating sensing-based micro-vibration sensor. The optical fiber has been directly treated as an elastomer to design the micro-vibration sensor, which possesses two FBGs. The mass is fixed on the middle of the fiber, and the vertical vibration of the mass has been converted into the axial tension/compression of the fiber. The principle of the sensor has been introduced, and the experiment conclusions show that the sensor sensitivity is 2362 pm/g within the range of 200–1200 mm/s^2^, which is consistent with theoretical analysis sensitivity of 2532.6 pm/g, and it shows an excellent linearity of 1.376%, while the resonant frequency of the sensor is 34 Hz, and the flat frequency range resides in the 0–22 Hz range. When used to measure micro-vibrations, its measured frequency relative error is less than 1.69% compared with the values acquired with a MEMS accelerometer, and the amplitude values of its measured vibration signal are consistent with the MEMS accelerometer under different excitation conditions too, so it can effectively realize the micro-vibration measurements.

## 1. Introduction

Compared with traditional electronic vibration sensors, fiber Bragg grating (FBG) sensing offers many advantages, for instance, immunity from electromagnetic interference and high temperatures, small size, corrosion resistance and ability to multiplex, so accelerometer design methods based on FBG are particularly attractive. Many scholars have done an in-depth research on them. Berkoff *et al.* proposed a new accelerometer, where the mass rested on a compliant material supported by a rigid base plate, and FBG embedded in the compliant material, using the mass’s movement conversion of the axial strain of the FBG by the compliant material [1]. Au *et al.* presented a tapered plate FBG accelerometer, where the FBGs have been pasted on the surface of the tapered plate; the experiment shows that its sensitivity is 18.93 με/g, and the maximum input signal frequency is up to 150 Hz [2]. A flexural beam utilized as primary transduction mechanism for demonstrating a FBG accelerometer has been described in [3]; it has good acceleration sensitivity of 212.5 με/g, and the resonant frequency is on the order of 1 kHz. Basumallick *et al.* proposed a method to improve the sensitivity of cantilever-mass-based FBG accelerometers by altering the distance between the axis of the FBG sensor to the neutral axis of the cantilever [4], and it’s demonstrated that its sensitivity is about 1062 pm/g. All of the above FBG vibration sensors’ principles are based on pasted FBGs, so these sensors’ sensing properties are limited by the pasting process and the elastomer structure.

Antunes *et al.* described the implementation and testing of an optical fiber-based accelerometer with cross axis insensitivity; its principle is based on an improved cantilever beam, and vertical vibration is converted into tension and compression movement along the FBGs’ axial direction by dangly arranged FBGs [5]. A *L*-shaped modified cantilever beam FBG-based accelerometer with self-temperature compensation has been studied in [6]; its sensitivity is 46 pm/g for frequencies below 50 Hz and 306 pm/g for frequencies above 150 Hz. da Costa Antunes *et al.* designed an accelerometer, where the inertial mass, supported by a *L*-shaped aluminum cantilever beam, was connected to the structure base by a steel leaf spring; when exposed to an external acceleration, the inertial mass’s movement along the vertical direction was converted into a contraction/expansion of the optical fiber through the *L*-shaped beam; it can be used to monitor structures with frequencies up to 45 Hz [7]. Reference [8] described the implementation and testing of an optical fiber biaxial accelerometer based on four FBGs placed in opposite positions; this is a simple solution to measure acceleration in two orthogonal directions, and its sensitivities were 87.848 and 92.351 pm/g, for each sensitive direction with resonant frequencies of 846.01 and 845.33 Hz, respectively. A fiber Bragg grating single-axis acceleration sensor based on a double-membrane has been proposed in [9]; its cross coupling of non-directional accelerations is minimized by introducing a unique double-membrane fixture of the sensor's mass of inertia leading to an almost diagonal form of the sensors stiffness-matrix; according to FEM simulation of the sensor, its resonance frequency is 6.0 kHz with a sensitivity of 1 pm/g. The references [10,11] proposed a non-contact vibration sensor based on fiber Bragg gratings, mainly used to measure displacement vibrations; the diaphragm is used as the elastomer, and its principle is similar to that described in [9]. Zhang *et al.* presented a novel FBG accelerometer, where the elastomer is the fiber itself, and the mass is fixed on the middle of the two FBGs to sense the vibration; it has good flat response from 10–130 Hz with a sensitivity of 231.8 pm/g [12]. A metal bellows-based FBG accelerometer is proposed and experimentally demonstrated in [13], the mass is also directly fixed on the fiber, the principle of the it is similar to the sensor in reference [12]; its sensitivity is 548.7 pm/g within a wide frequency response range 5–110 Hz. Guo *et al.* presented a fiber Bragg grating-based accelerometer with a fully metalized package, the elastic coefficient of fiber is greatly improved by this processing, the experiments shows that its resonant frequency is 3.6 kHz, and the sensitivity is 1.7 pm/g within the range of 0–8 g [14]. Based on the above investigation, the sensitivity of previous FBG accelerometers is commonly relatively lower. And all of these aren’t suited to measure micro-vibrations. Li *et al.* used the transverse property of optical fiber to design a triaxial vibration sensor; its sensitivity in the y direction is 971.8 pm/g, which is larger than the above designed FBG vibration sensors [15], but in [15], only experiments were used to verify the triaxial vibration measurement theoretical model, and it lacks a rigorous sensitivity theoretical model, so this paper mainly proposes a FBG-based vibration sensor to monitor micro-vibrations and builds an effective model for the sensitivity, which meets the micro-vibration requirements.

In this paper, a micro-vibration sensor based on FBG sensing has been proposed. The optical fiber has been directly treated as an elastomer, which possesses two FBGs, and the vertical vibration of the mass has been converted into the axial tension/compression of FBG to achieve the micro-vibration measurement. The principle of the sensor and experimental analyses are introduced.

## 2. Model and Principle of Micro-Vibration Sensor

**Figure 1:**
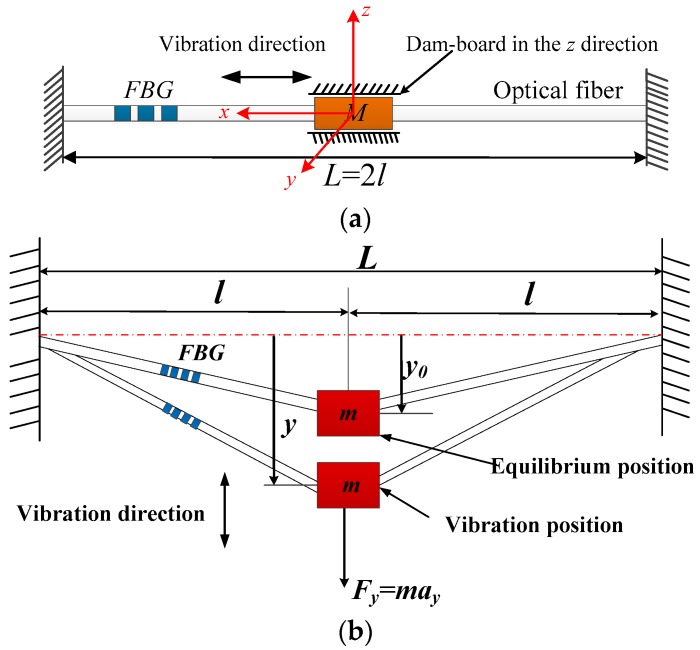
Vibration model of a bare optical fiber with FBG and lumped mass. (**a**) Horizontal vibration model of a bare optical fiber with FBG and lumped mass; (**b**) Vertical vibration model of a bare optical fiber with FBG and lumped mass.

Commonly designed FBG vibration sensors mainly use the mechanical structure as elastomer, such as the cantilevers in [6,7]. This paper directly uses the optical fiber as elastomer to design the vibration sensor (Figure 1), the *z* direction freedom of mass is limited by the dam-board. Its structure is very small and simple; also it’s easily to achieve quasi-distributed vibration measurements. According to the mechanics of the material, the horizontal direction stiffness *K_x_* of sensor can be expressed as:

(1) $$K_{x}=\frac{2E_{f}A_{f}}{l}$$

where *E_f_* is the Young’s Modulus of optical fiber, and *A_f_* means the cross-sectional area of the optic fiber. According to the material mechanics, the lateral bending stiffness of the fiber can be described as *K_L_* = *E_f_I_f_* = π*E_f_D^4^*/64. Young’s Modulus *E_f_* and diameter of optical fiber D are 72 GPa and 125 μm, respectively.

The lateral bending stiffness of fiber *K_L_* is equal to 8.63 × 10^−5^ N/m, which is close to 0, so the optical fiber can be considered as a string. Its lateral vibration model can be simplified as shown in Figure 1b. Combining with geometrical deformation method, the strain increment of the fiber are separately Δ*ε*_0_ and Δ*ε_y_* under mass in the equilibrium position or vibrating position, which can be expressed by:

(2) $$\Delta \varepsilon_{0}=\frac{\sqrt{l^{2}+y_{0}^{2}}-l}{l}$$

(3) $$\Delta \varepsilon_{y}=\frac{\sqrt{l^{2}+y^{2}}-l}{l}$$

where *y*_0_ represents the distance between the equilibrium position of the mass and a horizontal line, *y* is the vertical direction movement of inertial mass, *L*(*L* = 2*l*) is the initial length of the optical fiber between both fixed ends, *a_y_* is the acceleration along the *y* direction.

Pre-stress is exerted on the optical fiber during the sensor packaging process; the corresponding pre-strain of the fiber is *ε*_0_. Combining the equation of static theory with Equation (1), when the mass is in the equilibrium position, the gravity of mass *mg* can be represented by:

(4) $$mg=\frac{2E_{f}A_{f}(\varepsilon_{0}+\Delta \varepsilon_{0})}{(\Delta \varepsilon_{0}+1)l}y_{0}$$

where Δ*ε*_0_ is the strain increment under the inertial mass gravity, *g* represents acceleration of gravity.

Combining with Equation (4), the stiffness of optical fiber *K_y_* along the vibration direction can be written as:

(5) $$K_{y}=\frac{mg}{y_{0}}=\frac{2E_{f}A_{f}(\varepsilon_{0}+\Delta \varepsilon_{0})}{(\Delta \varepsilon_{0}+1)l}$$

Combining Equation (1) with Equation (5), the *R_x/y_* is the stiffness ratio between the horizontal and vibration direction stiffness, and it can be described by:

(6) $$R_{x/y}=\frac{\Delta \varepsilon_{0}+1}{\varepsilon_{0}+\Delta \varepsilon_{0}}\approx \frac{1}{\varepsilon_{0}+\Delta \varepsilon_{0}}$$

**Figure 2:**
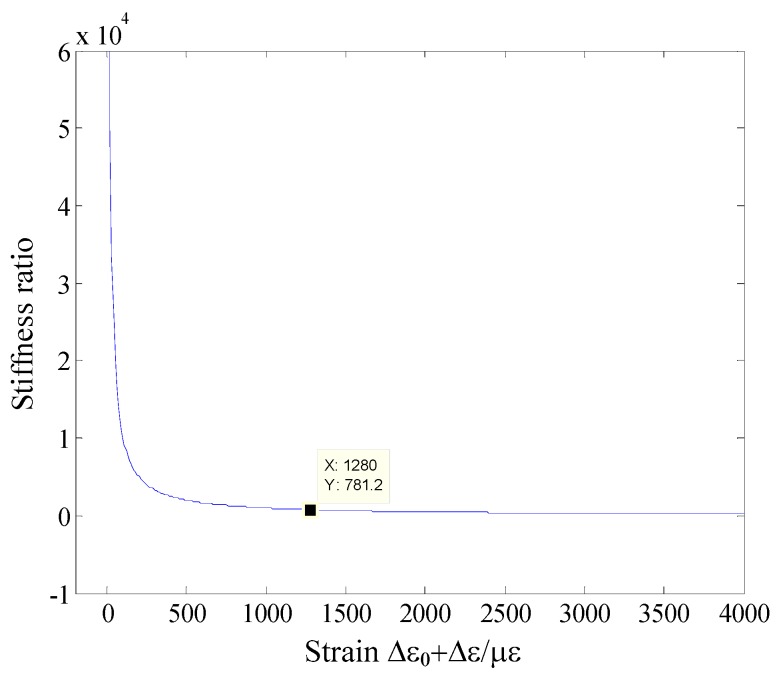
The stiffness ratio *versus* Δ*ε*_0_ + *ε*_0_.

From Equation (6), the stiffness ratio *versus* Δ*ε*_0_ + *ε*_0_ is shown in Figure 2. From Figure 2, the *R_x/y_* is far more than 1, which means the *K_x_* >> *K_y_*. The sensitivity of the vibration sensor is inversely proportional to the stiffness. The FBG vibration sensor was designed using the axial feature of optical fiber in [13], and the mass is 17.9 g, while the sensor’s sensitivity is only 231.48 pm/g. When the vertical direction of optical fiber is considered as the vibration direction, it will greatly improve the sensitivity of the FBG vibration sensor to use the same mass as reference [13]. Therefore, the vertical vibration property can be used to design a micro-vibration sensor.

Combining Equation (4) with the definition of resonant frequency, the resonant frequency *w_v_* of vibration direction can be written as:

(7) $$w_{v}=\sqrt{\frac{K_{y}}{m}}=\sqrt{\frac{2E_{f}A_{f}(\varepsilon_{0}+\Delta \varepsilon_{0})}{(\Delta \varepsilon_{0}+1)lm}}$$

When the excited vibration frequency *w* << *w_v_*, through the vibration mechanics, the relationship between *y* − *y*_0_ and acceleration *a_y_* can be expressed as:

(8) $$y-y_{0}=\frac{a_{y}}{w_{v}^{2}}$$

**Figure 3:**
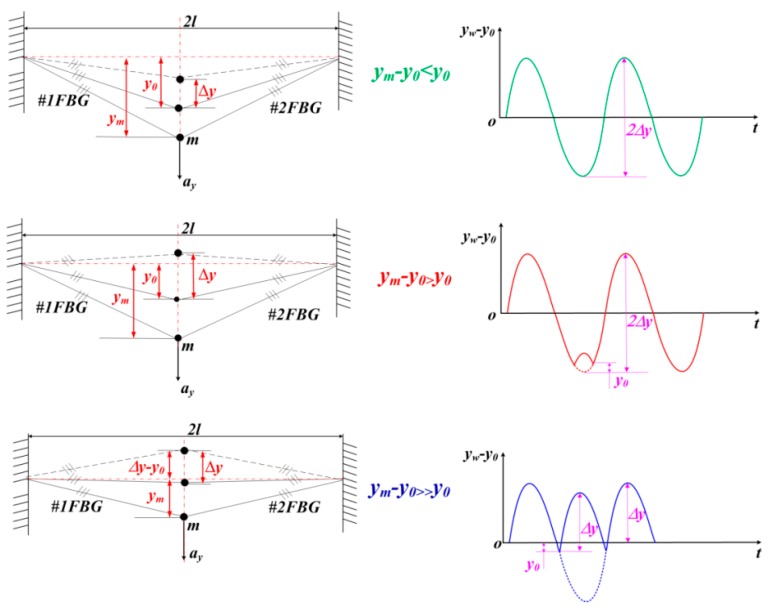
Response wave pattern for vertical vibration model of optical fiber.

The response wave pattern for the vertical vibration model of optical fiber is shown in Figure 3. In order to get a perfect vibration wave, it should satisfy the situation *y_m_* − *y*_0_ < *y*_0_ (where *y_m_* represents the maximum vibration deformation in the vertical direction) from Figure 3. When the vibration is very small, the relation between *y*_0_ and Δ*ε*_0_ can be simplified as linearity. Therefore, only if the maximum strain of FBG is less than Δ*ε*_0_ could the sensor accurately obtain the vibration signal. From this conclusion, we can effectively obtain the sensing ranges of the micro-vibration sensor.

Combining the Equation (2) with Equation (3), Equation (8) can be simplified as:

(9) $$\sqrt{{\left(\Delta \varepsilon_{y}+\Delta \varepsilon_{0}\right)}^{2}+2(\Delta \varepsilon_{y}+\Delta \varepsilon_{0})}=\sqrt{\Delta \varepsilon_{0}^{2}+2\Delta \varepsilon_{0}}\frac{a_{y}}{g}+\sqrt{\Delta \varepsilon_{0}^{2}+2\Delta \varepsilon_{0}}$$

From Equation (9), the acceleration *a_y_* can be expressed as:

(10) $$\frac{a_{y}}{g}+1=\frac{\sqrt{{\left(\Delta \varepsilon_{y}+\Delta \varepsilon_{0}\right)}^{2}+2(\Delta \varepsilon_{y}+\Delta \varepsilon_{0})}}{\sqrt{\Delta \varepsilon_{0}^{2}+2\Delta \varepsilon_{0}}}$$

According to the Taylor formula, Equation (10) can be expanded at the Δ*ε_y_* = 0. Due to the fact Δ*ε_y_* is very small, we extract the first order Taylor expansion. The acceleration *a_y_* can be rewritten as:

(11) $$a_{y}=\frac{4E_{f}^{2}A_{f}^{2}{\left(\varepsilon_{0}+\Delta \varepsilon_{0}\right)}^{2}}{m^{2}g(\Delta \varepsilon_{0}+1)}\Delta \varepsilon_{y}$$

**Figure 4:**
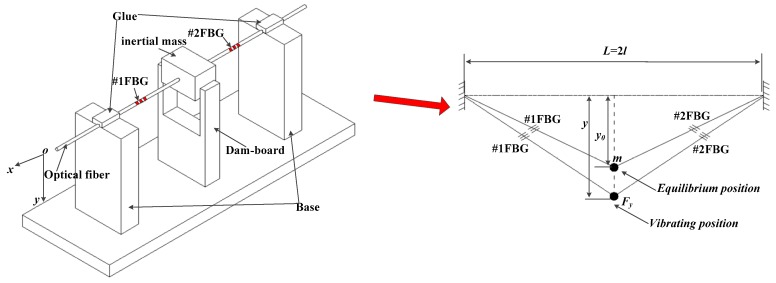
Schematic diagram of the FBG based micro-vibration sensor.

A schematic diagram of the FBG-based micro-vibration sensor is shown in Figure 4. The optical fiber has been directly treated as an elastomer. It has two FBGs (#1FBG and #2FBG), which can be used to eliminate the interference from the vertical vibration direction and enhance the sensitivity by adding the two FBGs’ center wavelength shift. The two sides of the fiber are fixed on the base with glue, and the mass is fixed on the middle of the fiber. When the mass moves along the vertical direction, it will cause a contraction/expansion of the FBGs, finally indicing the two FBGs’ wavelengths shift, so the vibration will be obtained by the FBGs’ center wavelength shift.

Assume the micro-vibration sensor is working at constant temperature, so according to the basic principle of FBGs, the center wavelength shifts of #1FBG and #2FBG are separately defined as:

(12) $$\begin{array}{c} \frac{\Delta \lambda_{1}}{\lambda_{1}}=(1-\rho_{e})\varepsilon_{1}\\ \frac{\Delta \lambda_{2}}{\lambda_{2}}=(1-\rho_{e})\varepsilon_{2} \end{array}\}$$

where λ_1_and Δλ_2_ represent the center wavelength of #1FBG and #2FBG, respectively; *ρ_e_* represents the strain-optic coefficient of the optical fiber; *ε*_1_ and *ε*_2_ are the axial strain of #1FBG and #2FBG, respectively.

As a result of the existence of machining errors and assembly errors, when the sensor is working, there always occurs some vibration interference from the horizontal direction, which isn’t limited (Figure 1), so #1FBG will be compressed or stretched under the inertial force of the horizontal direction, but the #2FBG is in the opposite state. The strain of the #1FBG and #2FBG are separately Δ*ε_x_* and −Δ*ε_x_* at any time. When the optical fiber moves under an inertial force in the vertical direction, both #1FBG and #2FBG will be compressed or stretched. Therefore, the strain increment of #1FBG Δ*ε_y_* is equal to the strain of #1FBG. Combining with Equation (12), the two FBGs’ center wavelengths can be rewritten as:

(13) $$\begin{array}{c} \frac{\Delta \lambda_{1}}{\lambda_{1}}=(1-\rho_{e})\varepsilon_{1}=(1-\rho_{e})(\Delta \varepsilon_{y}+\Delta \varepsilon_{x})\\ \frac{\Delta \lambda_{2}}{\lambda_{2}}=(1-\rho_{e})\varepsilon_{2}=(1-\rho_{e})(\Delta \varepsilon_{y}-\Delta \varepsilon_{x}) \end{array}\}$$

Since λ_1_, λ_2_ >> Δλ_1_, Δλ_2_ and λ_1_ ≈ λ_2_, combining Equation (13), the addition value Δλ_2_ + Δλ_1_ can be expressed as:

(14) $$\Delta \lambda_{1}+\Delta \lambda_{2}=(1-\rho_{e})\lambda_{1}\frac{m^{2}g(\Delta \varepsilon_{0}+1)}{2E_{f}^{2}A_{f}^{2}{\left(\varepsilon_{0}+\Delta \varepsilon_{0}\right)}^{2}}a_{y}$$

From Equation (14), Δλ_2_ + Δλ_1_ is only affected by *a_y_*, and the vibration interference from the *x*-direction is eliminated by the sum of two FBGs’ center wavelength shift. Also the sensitivity of the sensor is enhanced two times compared with the single FBG in Equation (11), which can be expressed by:

(15) $$S_{y}=(1-\rho_{e})\lambda_{1}\frac{m^{2}g(\Delta \varepsilon_{0}+1)}{2E_{f}^{2}A_{f}^{2}{\left(\varepsilon_{0}+\Delta \varepsilon_{0}\right)}^{2}}$$

According to the Equation (15), the micro-vibration can be obviously obtained by the two FBGs’ center wavelength shift.

## 3. Sensing Characteristic Experiments and Discussion

**Figure 5:**
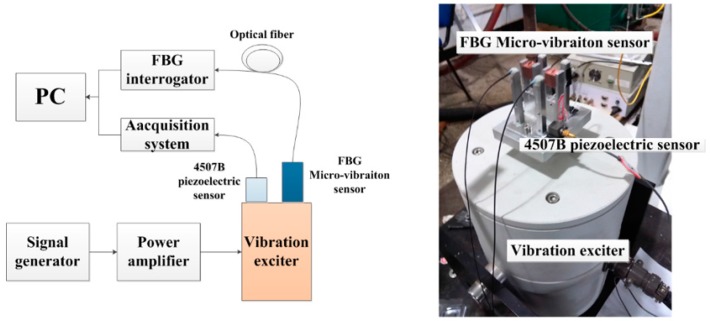
Schematic diagram and physical map of the experiments to determine the sensors’ sensing properties.

A schematic diagram and physical map of the experimental system is shown in Figure 5. The vibration exciter is driven by a signal generator and power amplifier. Both the FBG micro-vibration sensor and a 4507B piezoelectric sensor (sensitivity: 9.91 mv/ms^−2^) are fixed on the vibration exciter. The 4507B piezoelectric sensor is used as reference standard. The center wavelength signal of the FBGs and voltage signal are separately sent to a FBG interrogator (sample rate: 4 kHz; resolution ratio: 0.1 pm) and acquisition system. The FBG interrogator’s peak fluctuation value is about 5–6 pm without input excitation.

In this experiment, the mass is 8.93 g, the initial center wavelength of the two FBGs (length of FBG: 3 mm; reflectivity >90%) are separately 1298.226 and 1310.027 nm. During the processing of the sensor, at first, we use the optical fiber to connect with the mass, and then exert an appropriate pre-stress on the fiber. The FBGs’ center wavelengths shift is about 1.45 nm compared with the initial center wavelength. Initial length of the optical fiber *L* is 30 mm. Combining Equation (7) with Equation (15), the natural frequency and sensitivity of sensor can be calculated, which are separately 21.9 Hz and 2532.5 pm/g.

### 3.1. Sensitivity Experiments

**Figure 6:**
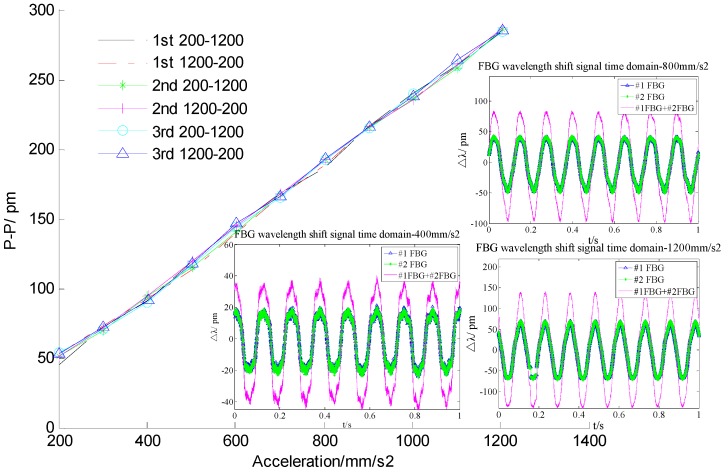
The addition value Δλ_2_ + Δλ_1_ *versus* applied acceleration *a_y_*—the inset shows the center wavelength shift response in the time domain with *a_y_* of 400 mm/s^2^, 800 mm/s^2^ and 1200 mm/s^2^.

**Figure 7:**
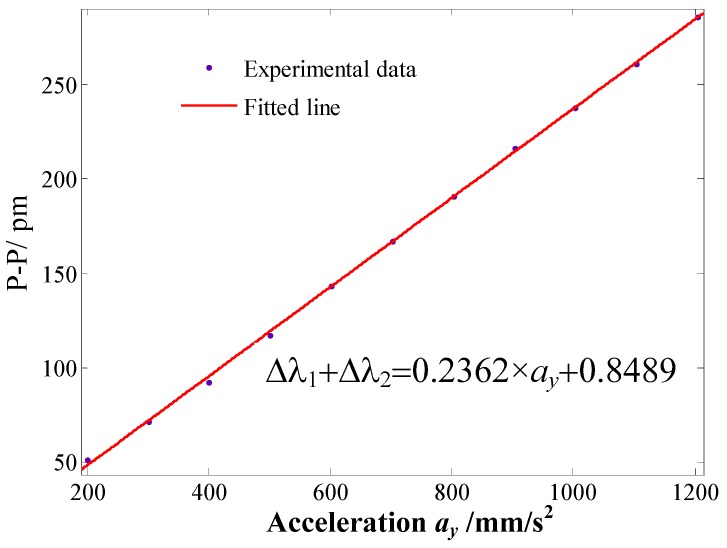
The linear fitting curve of addition value Δλ_2_ + Δλ_1_ *versus* applied acceleration *a_y_*.

During the experiment, the acceleration amplitude changes from 200 mm/s^2^ to 1200 mm/s^2^ under a constant frequency of 8 Hz through adjusting the signal generator. The experiment is repeated six times with the amplitude of acceleration being first increased and then decreased to demonstrate the repeatability and hysteresis of the sensor. According to Equation (14), the plot of the addition value Δλ_2_ + Δλ_1_ *versus* applied acceleration *a_y_* is shown in Figure 6. From Figure 6, the micro-vibration sensor’s repeatability error and hysteresis error can be obtained separately as 3.247% and 3.589%. In order to further study the sensor’s sensing properties, we average the six sets of experimental data, and then obtained a linear fitted curve of the sensor, which is shown in Figure 7. From Figure 7, we can get that the linearity is 1.376%; the fitted equation can be expressed as Δλ_2_ + Δλ_1_ = 0.2362 × *a_y_* + 0.8489 (unit of *a_y_* is mm/s^2^). The experimental sensitivity of 2362 pm/g is consistent with the theoretical analysis sensitivity of 2532.6 pm/g, so the validity of sensor’s theoretical sensitivity mode is verified. Its sensitivity is greatly increased compared with the sensitivity of the devices described in [13,14,15].

### 3.2. Amplitude-Frequency Property Experiments

**Figure 8:**
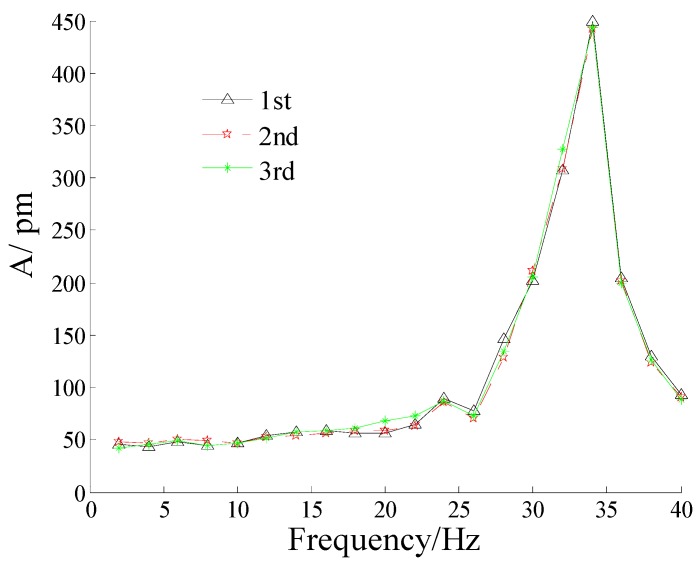
Amplitude-frequency curve of the FBG micro-vibration sensors in the *y*-direction.

In order to demonstrate the sensor’s amplitude-frequency properties, the acceleration amplitude is set at 400 mm/s^2^, the frequency increases from 2 to 40 Hz. Figure 8 shows amplitude-frequency curve of the FBG micro-vibration sensor. Figure 8 shows that sensor’s resonance frequency is about 34 Hz, and it has a flat response within 2 to 22 Hz. The experimental resonance frequency is larger than the theoretical computed value of 21.9 Hz. Because the effect of mass’s dimension is neglected in the theoretical model, and the equivalent stiffness of the sensor is less than in the real situation.

**Figure 9:**
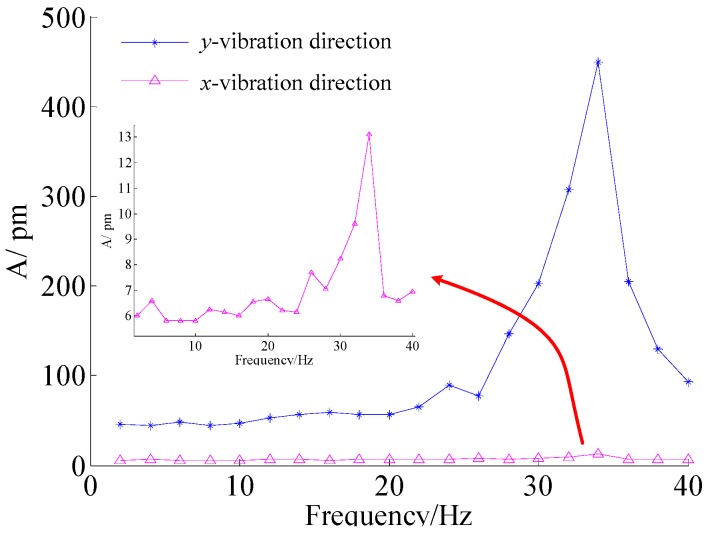
Amplitude-frequency curve of the FBG micro-vibration sensors in the *x* and *y*-direction.

In order to study the cross sensitivity of the sensor, we tried the *x* direction of the sensor as the excitation direction, and repeated the above experiment. Combining Figure 8, the amplitude-frequency curves of the FBG micro-vibration sensor in the *x* and *y*-direction are shown in Figure 9. From Figure 9, we can find that the pink curve is almost parallel to the *x* axis without the frequency of 34 Hz, which represents the cross interference response of the sensor; the amplitude of the pink curve is about 6 pm, mainly caused by the FBG interrogator. The cross interference of the sensor can be decreased by the addition of two FBGs by about 0.51% = (6 × 2/2362).

### 3.3. Experiments of Dampling Characteristics

The damping ratio is one of the most important indicators to evaluate the performance of the sensor, because it can be used to optimize the sensors’ structure. According to the theory of vibration, the damping ratio of sensor can be obtained through the logarithmic damping coefficient method in the hammer experiment. The logarithmic damping coefficient can be expressed as:

(16) $$\delta =\frac{1}{n}\text{ln}\frac{x_{1}}{x_{n+1}}=\frac{1}{n}\ln(\frac{e^{-\xi w_{v}t}}{e^{-\xi w_{v}(t+nT_{d})}})=\xi w_{v}T_{d}=\frac{2\pi \xi }{\sqrt{1-\xi^{2}}}$$

where *x*_1_ is the 1st reference peak value in the time domain, *x_n_*_+1_ is the nth reference peak value in the time domain, *ξ* means damping ratio, *T_d_* is period of vibration. According to Equation (16), the damping ratio can be written as:

(17) $$\xi =\frac{\delta }{\sqrt{4\pi^{2}+\delta^{2}}}$$

**Figure 10:**
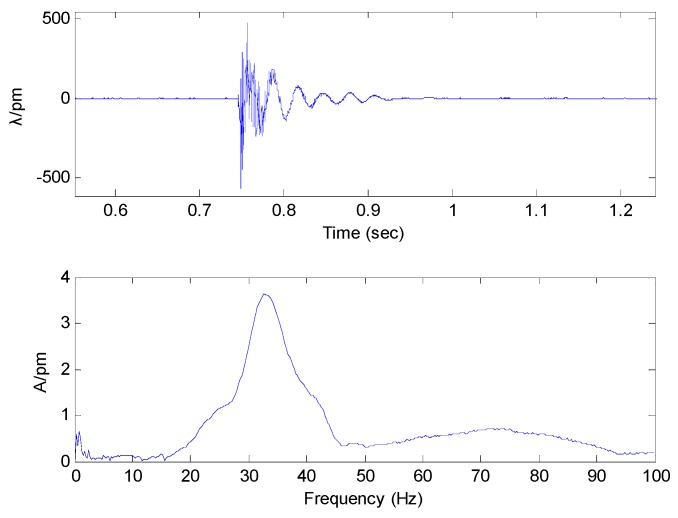
Time domain and spectrum of the micro-vibration sensor.

The FBG micro-vibration sensor is fixed on the table, and using the hammer we knock the table along the vertical direction. The time domain and spectrum of the micro-vibration sensor is shown in Figure 10. Combining Equations (16) and (17), the damping ratio of the FBG micro-vibration sensor is about 0.08955. The damping ratio of the vibration sensor is very small because its damping is mainly composed of optical fiber structural damping. Also from the Figure 10, we can get that sensors’ resonance frequency is about 34 Hz, which is consistent with the result from Figure 8.

## 4. Micro-Vibration Measurement Experiments and Discussion

**Figure 11:**
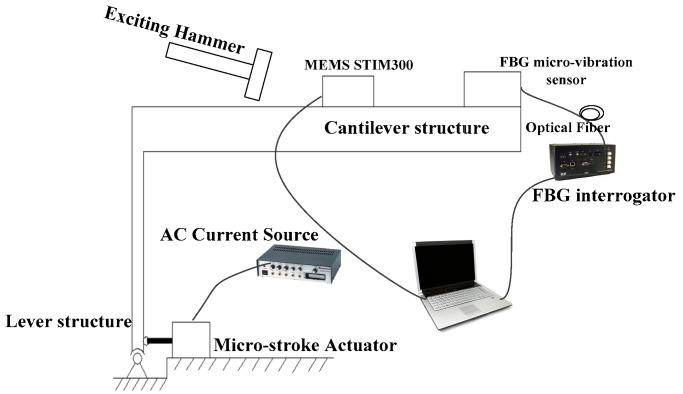
The principle diagram of the micro-vibration simulation experiment system.

In order to further evaluate the performance of the FBG micro-vibration sensor, several comparative tests are carried out based on the micro-vibration experiment system. The principle diagram of the micro-vibration experiment system is shown in Figure 11. This experimental system consists of a micro-stroke actuator, cantilever structure and lever structure, *etc.* The movement range of the micro-stroke actuator is from 100 nm to 100 μm. The cantilever structure is mainly used to amplify the micro-stroke actuator’s displacement, and convert horizontal micro-displacements into vertical vibrations of the cantilever structure. Both a MEMS vibration sensor (range: ±10 g; revolution:1.9 μg) and FBG micro-vibration sensor are fixed on the cantilever structure (Figure 11), The MEMS vibration sensor is taken as reference. In order to avoid any interference between the two sensors, there is a small distance between them.

### 4.1. Experiments of Hammering Excitation

**Figure 12:**
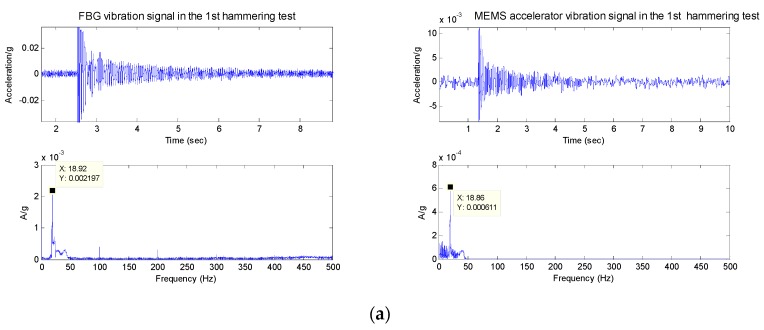

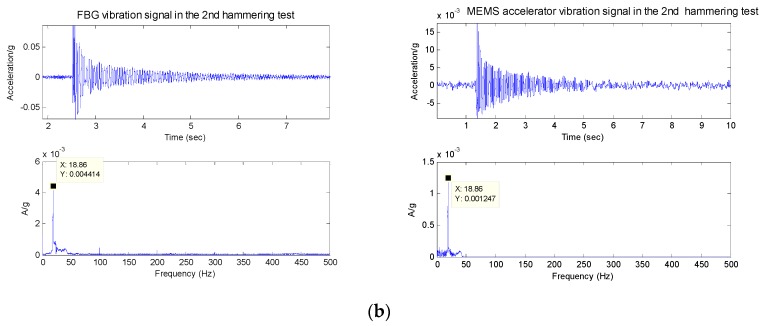
The responsivity of the two kinds of sensors’ signals for the hammering excitation experiment. (**a**) The time domain and spectrum of the two kinds of sensors for the 1st hammering excitation experiment; (**b**) The time domain and spectrum of the two kinds of sensors for the 2nd hammering excitation experiment.

Using the hammer to hit the root of the cantilever, the hammering excitation signal is simultaneously acquired by the MEMS and FBG micro-vibration sensors. Due to the fact the hammering excitation can be simplified as a pulse signal, the resonant frequency can be obtained from the response signals of the two kinds of sensor. Figure 12 shows the response signals of the two kinds of sensors under the hammering excitation. Figure 12 reveals that for time domain signals, the tendency of the MEMS and FBG micro-vibration sensor is almost the same. The resonant frequency of the micro-vibration experiment system can be estimated from the signals obtained from the two kinds of sensors; both experimental results are same, which is about 18.86 Hz (Table 1). This hammering excitation test also confirms the performance of FBG micro-vibration sensor is good.

### 4.2. Scanning Frequency Experiments

**Figure 13:**
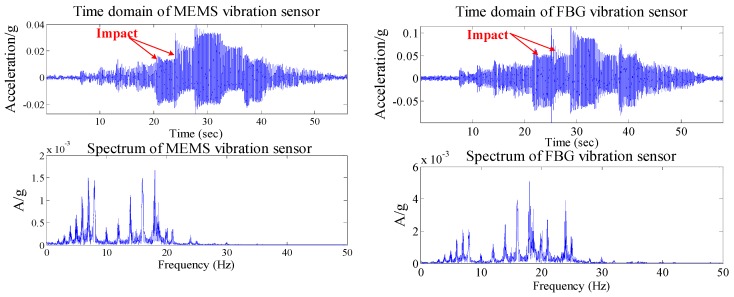
Time domain and spectrum map of the MEMS and FBG micro-vibration sensors for the scanning frequency experiments within 1–8 Hz.

In order to study in depth the performance of the FBG micro-vibration sensor, scanning frequency experiments have been done in this part. The current amplitude is a constant, the frequency increases from 1 to 8 Hz, and interval is set at 1 Hz. The time interval between each frequency was kept at about 3~4 s by manual operation. The micro-stroke actuator has been driven by the above electrical signals. The time domain and spectrum maps of the MEMS and FBG micro-vibration sensors for the scanning frequency experiment within 1–8 Hz are shown in Figure 13. For the time domain map of the two sensors, the same conclusion of the hammering excitation experiment can be reached. When the excitation frequency is changed, it generates at impact phenomenon in Figure 13, so we can determine the changed time of the frequency through the impact phenomenon in Figure 13.

The frequency components of the MEMS and FBG micro-vibration sensors can be obtained from the spectrum maps in Figure 13, which are shown in Table 2. From Table 2, we can get that: the collected frequency components of the FBG micro-vibration sensor are consistent with the MEMS vibration sensor; for the 1× components, it’s consistent with the MEMS vibration sensor. The FBG micro-vibration sensor is more sensitive to high-frequency components; it can get more frequency multiplication. This may be caused by the structure itself or the measured body, but on the whole the FBG micro-vibration sensor can effectively measure the vibration, as well as display good performance.

### 4.3. Accuracy Comparison Experiments

**Figure 14:**
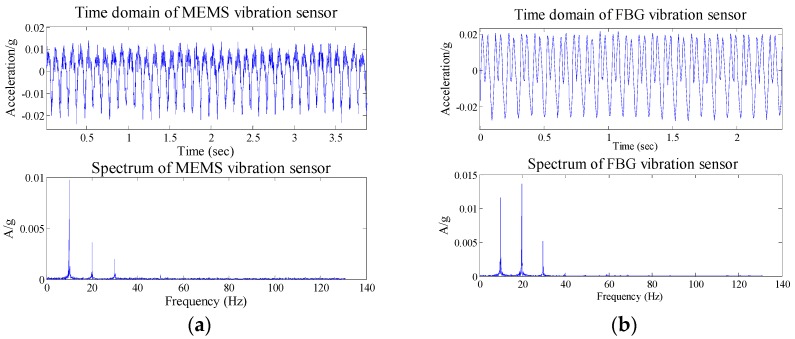
Time domain and spectrum map of the two sensors with s frequency of 10 Hz and current of 0.8 A. (**a**) Time domain and spectrum map of the MEMS vibration senor; (**b**) Time domain and spectrum map of the FBG micro-vibration senor.

To study the measurement accuracy of the FBG vibration sensor, we adjust the micro-stroke actuator incentive stroke work to different frequencies or current amplitudes. Figure 14 shows the time domain and spectrum map of the two sensors at a frequency of 10 Hz and current of 0.8 A. From Figure 14, we see that the time domain signals of the two sensors are almost the same. There exists a mass of glitches in the MEMS vibration signal’s time domain map, but the signal of the FBG micro-vibration sensor is very smooth. Also the frequency multiplication characteristics of the FBG micro-vibration sensor are even more obvious compared with the MEMS vibration sensor.

**Figure 15:**
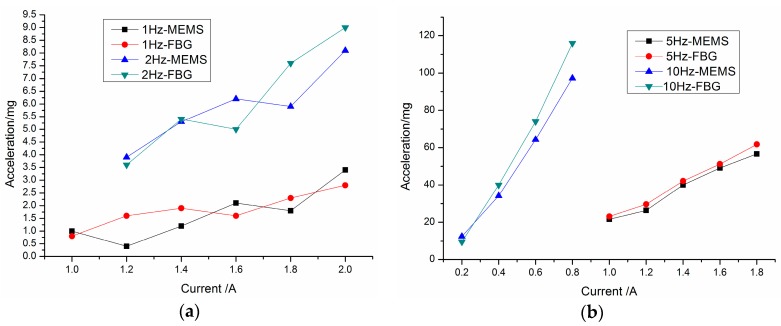
Comparison of the measured acceleration graphs of the two kinds of sensors under different amplitude of current with frequencies of (**a**) 1 Hz and 2 Hz; (**b**) 5 Hz and 10 Hz.

In order to further verify the measurement properties of the FBG micro-vibration sensor, the amplitude of the fundamental frequency has been chosen in Figure 15. It shows the measured acceleration of the two kinds of sensors under different current amplitudes with a frequency of 1 Hz, 2 Hz, 5 Hz and 10 Hz. From Figure 15, we can find that the measured acceleration amplitude of the FBG micro-vibration sensor is consistent with the MEMS vibration sensor. Also two other conclusions can be obtained: (1) There is no difference between the measured acceleration amplitudes of the two sensors at 1 Hz and 2 Hz; (2) When the excited frequencies are set at 5 and 10 Hz, the acquired signal amplitude of the FBG micro-vibration sensor is slightly larger than that of the MEMS vibration sensor. There are two main reasons that could responsible for this phenomenon: (i) the FBG micro-vibration sensor is fixed in place nearer to the end of the cantilever structure compared with the MEMS vibration sensor, so the relative error between the two sensors will be increased as the vibration amplitude increases; (ii) the fitted line is obtained under an excitation frequency of 8 Hz, although the FBG vibration sensor has a flat response between 2 to 22 Hz, there exists a small non-linearity from 6 to 22 Hz, and it’s not absolutely parallel to the *x* axis in the Figure 8. This causes the sensitivity to increase along with the excitation frequency growth. From the above analysis, the tendency of the two sensors’ signals are the same, which demonstrates that the FBG micro-vibration sensor can be applied to measure micro-vibrations, but the accuracy and reliability of the FBG micro-vibration sensor need to be further improved.

## 5. Conclusions

This paper has proposed a fiber Bragg grating sensing-based micro-vibration sensor. The optical fiber has been directly treated as an elastomer, and the mass is fixed on the middle of the fiber. The vertical vibration of the mass has been converted into the axial tension/compression of the fiber, and finally this deformation induces the same variations of the two FBGs’ wavelength. Adding the two FBGs’ center wavelength shift, the sensitivity has increased two-fold compared with the FBG signal and eliminated the interference from the *x*-direction. The principle of the sensor has been studied in this paper, and the experimental conclusions show that: (i) the sensitivity of the FBG micro-vibration sensor is 2362 pm/g within the range of 200–1200 mm/s^2^, which is consistent with the theoretical sensitivity of 2532.6 pm/g, illustrating that the theoretical sensitivity model of the sensor is correct; (ii) it shows an excellent linearity which is 1.376%; (iii) the resonant frequency of the sensor is 34 Hz, and the flat frequency range lies within 0–22 Hz. When used to measure micro-vibrations, the relative error of the measured frequency is less than 1.69% compared with a MEMS vibration sensor; and the amplitude values of its measured vibration signal are consistent with the MEMS accelerometer under different excitation conditions, too. This method has effectively improved the sensitivity compared with the traditional FBG vibration sensor, and it can be used to measure micro-vibrations with high precision.

## Figures and Tables

**Table 1:** The resonance frequency of micro-vibration structure obtained by the MEMS and FBG micro-vibration sensor.

Test Number	FBG Micro-Vibration Sensor	MEMS STM300
1	18.92 Hz	18.86 Hz
2	18.86 Hz	18.86 Hz

**Table 2:** Signals’ frequency components of the MEMS and FBG micro-vibration sensors for the scanning frequency experiments.

	MEMS	FBG Micro-Vibration Sensor
1× (Fundamental frequency)/Hz	0.9003/2.045/3.006/4.044/5.02/5.996/6.973/7.996	0.9155/2.09/3.006/4.004/5.02/5.996/6.973/7.996
Frequency multiplication/Hz	9.995/12.04/14.05/15.01/15.99/18.07/18.62/20.11/21.04/23.96/25.01/28.03/29.98/32.09	9.995/11.99/14.05/15.01/15.99/18.07/18.63/20.13/21.03/23.97/24.98/27.98/30/32.06/34.97/36.13/40.07
